# Lemmel Syndrome: Unveiling the Underrecognized Pancreatobiliary Diagnosis, Management Strategies, and Future Directions

**DOI:** 10.7759/cureus.61673

**Published:** 2024-06-04

**Authors:** Fnu Vikash, Sandesh Parajuli, Vikash Kumar, Sunny Patel, Sammy Ho

**Affiliations:** 1 Department of Internal Medicine, Albert Einstein College of Medicine, Jacobi Medical Center, New York City, USA; 2 Department of Medicine, Reading Hospital, Tower Health, West Reading, USA; 3 Department of Internal Medicine, The Brooklyn Hospital Center, New York City, USA; 4 Department of Gastroenterology and Hepatology, Albert Einstein College of Medicine, Jacobi Medical Center, New York City, USA; 5 Department of Gastroenterology, Albert Einstein College of Medicine, Jacobi Medical Center, New York City, USA

**Keywords:** lemmel syndrome, transaminitis, ampulla of the vater, periampullary diverticulum, double duct sign

## Abstract

Lemmel syndrome, a rare condition, is characterized by biliary obstruction caused by a periampullary diverticulum (a pouch-like outgrowth of the duodenum near the ampulla of Vater). In our case, a 76-year-old male patient presented with epigastric pain and exhibited a cholestatic pattern on liver function tests. Imaging revealed dilated pancreatic and common bile ducts due to compression by a periampullary diverticulum (double duct sign: simultaneous dilation of the common bile duct and pancreatic duct). Upper endoscopy showed one medium-sized periampullary diverticulum. This case emphasizes the diagnostic process and the importance of considering Lemmel syndrome in differential diagnosis in elderly patients with biliary obstruction. We discuss the prevalence, diagnostic considerations, including imaging modalities, and treatment options, emphasizing the need for further research.

## Introduction

Approximately 79% of small bowel diverticula were found in the duodenum, and the prevalence of duodenal diverticula is around 22% in cadaveric studies, which also increases with age [1,2]. Only 1-5% of duodenal diverticula are symptomatic, and common complications include bleeding, diverticulitis, and malabsorption [1,2]. A periampullary diverticulum (PAD), also known as a juxtapapillary diverticulum, is located within two to three centimeters of the ampulla of Vater [3]. However, when a PAD causes compression on the common bile duct (CBD) and/or pancreatic duct (PD), resulting in biliary obstruction, this condition is known as Lemmel syndrome [4]. It is a rare and underreported condition accounting for less than 1%, and there have only been several case reports since Dr. Gerhard Lemmel first described it in 1934 [5]. The highest differential for patients with biliary obstruction are cholelithiasis, benign biliary stricture, malignancy, and sclerosing cholangitis [6]. Very rarely do we find diverticulum as the cause of obstruction. Consequently, fewer clinicians may recognize Lemmel syndrome, posing a potential risk in clinical practice. We present a case of Lemmel syndrome to raise awareness among clinicians and emphasize its consideration as a rare differential diagnosis.

## Case presentation

**Figure 1:**
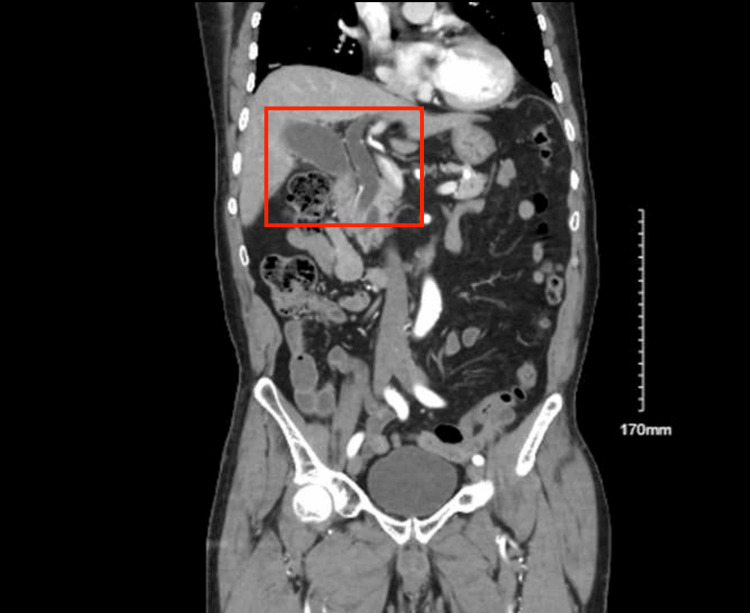
CT scan (coronal section) showing common biliary duct and pancreatic duct dilatation (~12 mm and 2 mm, respectively), secondary to periampullary diverticulitis-induced double-duct sign.

A 76-year-old male with a chronic history of peptic ulcer disease presented to the emergency department with epigastric pain for four days, aggravated by eating. He denies any nausea, vomiting, diarrhea, fever, and chills. He denies any family history of cancer and denies smoking, alcohol, or illicit drug use. Physical examination was remarkable for epigastric tenderness. Laboratory values showed a normal complete blood count (CBC). However, the liver function test demonstrated a cholestatic pattern with transaminitis and hyperbilirubinemia. The tumor markers, hepatitis panel, and HIV test were negative (Table 1). Computed tomography (CT) scan of the abdomen and pelvis with contrast showed a dilated PD and CBD, 2 mm and 12 mm, respectively with a hydropic gallbladder consistent with a double duct sign (Figure 1).

**Table 1:** Laboratory test results at the time of admission WBC: white blood cell; HGB: hemoglobin; PLT: platelet count; AST: aspartate aminotransferase; ALT: alanine aminotransferase; ALP: alkaline phosphatase; CEA: carcinoembryonic antigen; AFP: alpha-fetoprotein; CA-19-9: carbohydrate antigen 19-9; HBsAg: hepatitis B surface antigen; HBsAb: hepatitis B surface antibody; HbcAb: hepatitis B core antibody; HIV: human immunodeficiency virus

Laboratory Test	Result	Reference Range
WBC	6.78/nL	3.90-10.60/nL
HGB	14 g/dL	13.5-17.5 g/dL
Platelet count	149/nL	150-440/nL
ALP	199 U/L	40-129 U/L
ALT	182 U/L	0-40 U/L
AST	245 U/L	0-40 U/L
Total bilirubin (TB)	2 mg/dL	0.1-0.3 mg/dL
Direct bilirubin (DB)	2 mg/dL	0.1-0.3 mg/dL
Lipase	85 U/L	7-60 U/L
CEA	1.9 ng/mL	0.2-5.0 ng/mL
AFP	2.3 µg/L	0-9 µg/L
CA-19-9	17 U/mL	<35 U/mL
HBsAg	Non-reactive	-
HBsAb	Reactive	-
HbcAb	Non-reactive	-
Hepatitis A	Non-reactive	-
Hepatitis C	Non-reactive	-
Hepatitis E	Non-reactive	-
HIV test	Non-reactive	-

**Figure 2:**
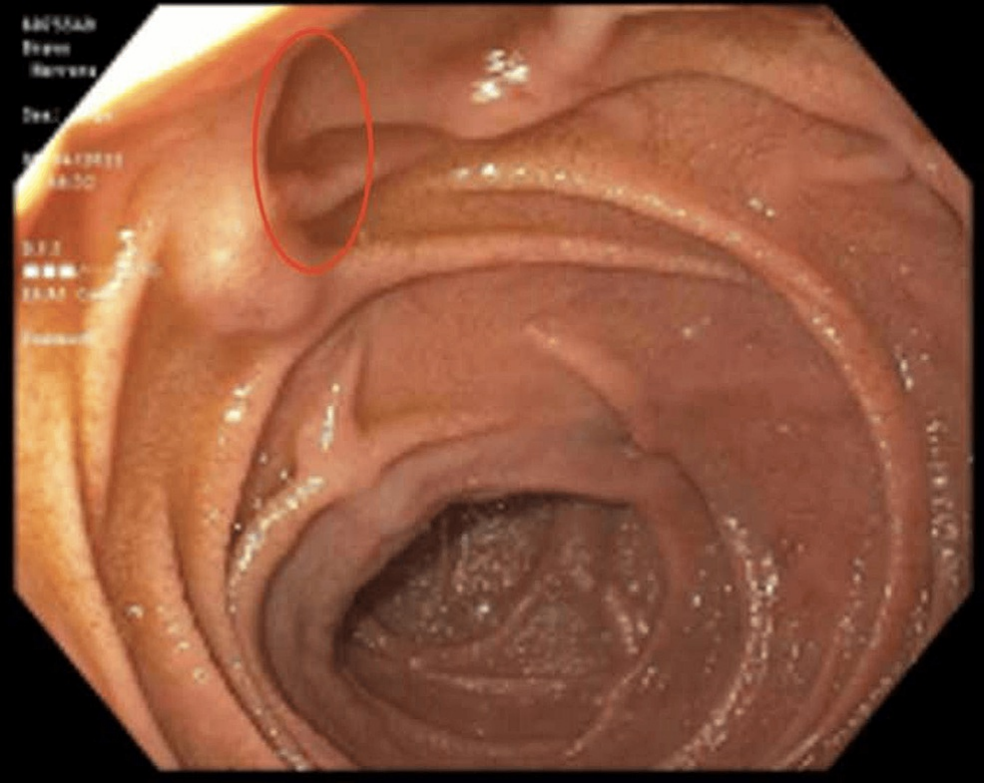
Upper endoscopy shows one medium-sized periampullary diverticulum (red circle) with debris vs stone compressing the ampullary duct without common biliary duct stone or tumor.

Upper endoscopy showed one medium-sized PAD with debris versus stone compressing the ampullary duct without CBD stones or tumors (Figure 2). On the upper endoscopy, there was erythematous mucosa found in the duodenum and stomach, with a biopsy showing chronic duodenitis and gastritis, respectively. The patient's symptoms resolved spontaneously and discharged with outpatient gastroenterology follow-up. However, unfortunately, the patient lost the follow-up and did not show up in the gastroenterology clinic.

## Discussion

Here, we presented an elderly male with Lemmel syndrome who was afebrile, and his only symptom was epigastric pain, which is common in patients with biliary obstruction. According to a literature review by Love et al., which summarized 17 subjects, 64.7% have abdominal pain, and 52.9% have fever [4]. Other symptoms include jaundice, weakness, fatigue, weight loss, and general deterioration. The mean bilirubin level was 5.9mg/dL, higher than we found in this patient. We speculate that the bilirubin level and symptoms could be determined by the distance between the edge of the diverticulum and ampulla, the presence of stone or debris in the diverticulum, the extent of obstruction, and the duration between symptom onset and clinical presentation. Most subjects received endoscopic intervention (46.7%) or surgical diverticulectomy (20%). However, our case did not receive aggressive treatment or antibiotics because of spontaneous improvement and the absence of fever and leukocytosis. Symptoms of Lemmel syndrome can spontaneously resolve due to the intermittent nature of the obstruction caused by the duodenal diverticulum. Changes in patient positioning, bowel motility, or the size of the diverticulum itself can temporarily relieve the obstruction, leading to a transient improvement in symptoms which could be one of the reasons in our case.

Lemmel syndrome is an uncommon cause of biliary obstruction, but PAD is common and occurs in >30% of patients aged more than 70 years [7]. Although duodenal diverticula are usually asymptomatic incidental findings in endoscopy or endoscopic retrograde cholangiopancreatography (ERCP), they are associated with increased risk for biliary conditions such as choledocholithiasis due to obstruction and biliary sludge formation, cholecystolithiasis, cholecystitis, cholangitis, and pancreatitis. For example, the risk of choledocholithiasis in patients with periampullary diverticula is doubled compared to those without periampullary diverticula [8]. A larger size (>2 cm) of periampullary diverticula is also associated with a higher risk of choledocholithiasis [9]. Therefore, in patients with biliary obstruction and known PAD without apparent stones, Lemmel syndrome should be higher in the differentials. Likewise, in patients with biliary obstruction without signs of stones, malignancy, or infection, Lemmel syndrome should also be considered. While abdominal ultrasound typically serves as the preliminary imaging technique, it may not always be informative. In cases of high suspicion, reviewing previous imaging and prioritizing CT scans or magnetic resonance cholangiopancreatography (MRCP) as first-line imaging modalities is recommended [10]. Another concern of the PAD is that it may act as a nesting area for bacterial growth, thus predisposing bacterial colonization of the bile duct. Bile stasis and contamination by beta-glucuronidase-producing bacteria can lead to the development of stones and biliary complications such as cholangitis. The high prevalence of periampullary diverticula raises concerns about elevated complication rates with ERCP. Bleeding and papillary edema due to frequent cannulation are common. Using a PD guidewire-indwelling technique may be beneficial to minimize these side effects [11,12].

## Conclusions

In conclusion, Lemmel syndrome remains a poorly understood condition due to its rarity. Larger scale studies on clinical characteristics are still needed to search for specific manifestations in the clinical diagnosis of Lemmel syndrome and to develop less invasive diagnostic tools. More data on the treatment and long-term outcomes are also needed to assess the necessity of diverticulectomy.
